# Immune Reconstitution Inflammatory Syndrome Related to Antiretroviral Therapy Initiation in People With HIV and Mpox: An Observational Retrospective Study

**DOI:** 10.1093/ofid/ofae374

**Published:** 2024-07-08

**Authors:** Juan Carlos Rodríguez-Aldama, Edgar Pérez-Barragán, Graciela Hernández-Silva, Jezer Ivan Lezama-Mora, Ana Karen Olin-López, Berenice González-Flores, Raul Adrián Cruz-Flores, Brenda Crabtree-Ramírez

**Affiliations:** Department of Infectious Diseases, Clínica Especializada Condesa Iztapalapa, Mexico City, Mexico; Department of Infectious Diseases, Clínica Especializada Condesa Iztapalapa, Mexico City, Mexico; Department of Infectious Diseases, Clínica Especializada Condesa, Mexico City, Mexico; Department of Infectious Diseases, Clínica Especializada Condesa, Mexico City, Mexico; Department of Infectious Diseases, Clínica Especializada Condesa Iztapalapa, Mexico City, Mexico; Department of Infectious Diseases, Clínica Especializada Condesa Iztapalapa, Mexico City, Mexico; Department of Epidemiology, Clínica Especializada Condesa Iztapalapa, Mexico City, Mexico; Department of Infectious Diseases, Instituto Nacional de Ciencias Médicas y Nutrición, Salvador Zubirán, Mexico City, Mexico

**Keywords:** IRIS, mpox, PWH, limited resources, early treatment

## Abstract

The study aims to compare the outcomes of initiating antiretroviral therapy early vs late in people with HIV and mpox. No worse outcomes were found associated with mpox-related immune reconstitution inflammatory syndrome among those who started antiretroviral treatment early, suggesting initiation as soon as possible.

The 2022 mpox outbreak mainly affected men who have sex with men, including those with HIV [1] and especially those with CD4+ cell counts <200 cells/mm^3^, who face heightened risks of complications. Mortality rates in individuals with late HIV presentation range from 15% to 40%, which suggest sustained viral replication in this population, favoring more severe disease [2, 3]. This evolution has led to mpox infection being considered an HIV-related opportunistic infection (OI) [4].

In contrast, cases have been reported of individuals with mpox and advanced HIV infection who started or restarted antiretroviral therapy (ART) and presented worsening and complications of mpox infection, as usually observed in paradoxical immune reconstitution inflammatory syndrome (IRIS). However, the mechanism behind this phenomenon is still not clear [5, 6]. The aim of the study was to report the findings of initiating ART early vs late in people with untreated HIV and mpox.

## METHODS

### Design

An observational retrospective study was conducted between June 2022 and January 2023 at Clínicas Especializadas Condesa Iztapalapa and Cuauhtémoc in Mexico City. Patients were followed from initial evaluation to clinical resolution or death.

### Study Population

This study enrolled people with HIV aged ≥18 years, including those who were treatment naive and those who had discontinued ART for >3 months. Inclusion criteria were based on the operational definition of mpox from the standardized procedures manual of Mexico [7] and guidelines of the World Health Organization [8]. We defined IRIS-related mpox as (1) a worsening of previously described symptoms and/or the appearance of new symptoms or (2) a >20% increase in previously identified lesions that could not be explained by any cause other than mpox.

Inclusion criteria required manifestation of 1 or more lesions consistent with mpox, accompanied by systemic signs and symptoms. Confirmation of mpox was achieved through a positive result in a real-time polymerase chain reaction test. Additionally, the study incorporated all patients who gave consent to initiate antiretroviral treatment, with this decision made at the discretion of the treating physician.

### Patient consent statement

All individuals provided written informed consent as part of routine care for suspected mpox cases at Clínicas Especializadas Condesa, involving the collection of samples and clinical data following established protocols for submission to the Ministry of Health of Mexico.

### Statistical Analysis

We detailed cases of IRIS-related mpox, including hospitalizations, deaths, retention in care, and other clinically relevant data. Results were stratified by early (<7 days) or late (>7 days) ART initiation post–mpox diagnosis.

Continuous variables were described as median (IQR) and categorical variables as counts and percentages. No imputation methods were applied to missing data. Analysis was done with SPSS (version 27, IBM), employing descriptive and comparative analyses, with the Mann-Whitney *U* test for nonparametric data and Fisher exact test for categorical data.

## RESULTS

Out of 689 cases of mpox identified in people with HIV, 19 cisgender men met the inclusion criteria: 14 (73.7%) had a previous HIV diagnosis without ART or with abandonment of ART, and 5 (26.3%) were treatment naive. The median CD4 cell count was 236 cells/µL (IQR, 64–339), with a median HIV-1 RNA of 74 334 copies/mL (IQR, 15 575–233 437). The demographics, epidemiologic characteristics, and outcomes of participants are stratified by time of ART initiation in Table 1.

**Table 1.** Baseline Demographic Data and Outcomes

	Total (n = 19)	Early ART <7 d (n = 10)	Late ART >7 d (n = 9)
Age, y	33 (28–41)	29.5 (25–35.7)	36 (32–45)
Cisgender men	19 (100)	10 (100)	9 (100)
HIV status			
Previously diagnosed without ART	14 (73.7)	6 (60)	8 (88.9)
Newly diagnosed	5 (26.3)	4 (40)	1 (11.1)
Time HIV diagnosis, y	0 (0–10)	0 (0–0.25)	8 (0.5–12)
Last CD4 count, cells/mm^3^	236 (64–339)	227.5 (98.5–285.5)	236 (46.5–425.5)
HIV-1 RNA viral load, copies/mL	74 334 (15 575–233 437)	69 559 (9193.7–201 124.5)	78 191 (18 129–251 466.5)
Additional conditions			
Hepatitis B	1 (5.3)	0 (0)	1 (11.1)
Hepatitis C	2 (10.5)	1 (10)	1 (11.1)
Syphilis	3 (15.8)	3 (30)	0 (0)
Anal warts	1 (5.3)	1 (10)	0 (0)
History of mpox vaccination	0 (0)	0 (0)	0 (0)
Mpox rash presentation			
Peak number of skin lesions	30 (20–50)	42.5 (22.5–50)	27.5 (15.5–82.5)
Rash duration, d	28 (20.2–43.7)	25 (18–37.5)	32.5 (22–48.7)
Severity skin score^a^			
Mild	5 (26.3)	2 (20)	3 (33.3)
Moderate	8 (42.1)	5 (50)	3 (33.3)
Severe	2 (10.5)	1 (10)	1 (11.1)
Very severe	1 (5.3)	0 (0)	1 (11.1)
Missing data	3 (15.8)	2 (20)	1 (11.1)
IRIS mpox	2 (10.5)	1 (10)	1 (11.1)
Outcomes			
Outpatient	17 (89.4)	10 (100)	7 (77.7)
Deaths	0 (0)	0 (0)	0 (0)
Hospitalization	2^b^ (10.5)	0 (0)	2 (22.2)
Lost to follow-up	2 (10.5)	1 (10)	1 (11.1)
Retention in care	17 (89.4)	9 (90)	8 (88.8)

Data are presented as No. (%) or median (IQR).

Abbreviations: ART, antiretroviral therapy; IRIS, immune reconstitution inflammatory syndrome.

^a^<25 lesions, mild; 25–99 lesions, moderate; 100–250 lesions, severe; >250 lesions, very severe.

^b^One individual was hospitalized for IRIS-related mpox, while another was hospitalized for suspected neuroinfection unrelated to mpox.

In terms of clinical characteristics, 10 participants (52.6%) experienced fever; 19 (100%) developed a skin rash; 15 (78.9%) presented vesicular-pustular lesions, with a median 30 lesions (IQR, 20–50); and 6 (31.6%) had >50 skin lesions attributable to mpox diagnosis in the baseline evaluation. Additionally, 2 (10.5%) presented coalescent-necrotic lesions.

The median time to start ART for all was 2 days (IQR, 1–33), with a median 1 day (IQR, 1–1) vs 33 days (IQR, 26–63.5) between those who received early and late ART. Twelve (63.2%) started ART before achieving clinical mpox resolution, and 10 (52.6%) began ART <7 days after mpox diagnosis. The median time to clinical mpox resolution was 25 days (IQR, 18–37.5) and 32.5 days (IQR, 22–48.7; not significant) between those who began early and late ART. The median CD4 cell count of those with early ART was 227.5 cells/µL (IQR, 98.5–285.5) vs 236 cells/µL (IQR, 46.5–425.5) for those with late ART. Eight patients (42.1%) had a CD4 count <200 cells/µL; 6 (31.6%) had a CD4 count <100 cells/µL; and 7 (36.8%) had an HIV RNA >100 000 copies/mL.

Of 19 individuals, 2 (10.5%) met the criteria for IRIS-related mpox: 1 each from the early and late ART initiation groups (not significant; Table 2). None received any antiviral against mpox. No deaths were identified, but 2 cases were lost to follow-up: 1 in the late ART start group with IRIS-related mpox and 1 in the early ART start group.

**Table 2.** People With HIV With IRIS Related to Mpox

Participant	Medical History	Initial Symptoms	Time to Initiation of ART After Diagnosis Mpox: Treatment	IRIS-Related Mpox Symptoms	Treatment and Outcome
Cis man; age, 46 y	HIV diagnosis 13 y ago, nonadherent to ART, stopped ART 35 wk ago. Latest HIV viral load: 99 272 copies/mL. CD4: 41 cells/mm³	Disseminated vesiculopustular lesions (30 lesions at its peak) with inguinal adenopathy	Day 72: FTC/TDF + DRV/COBI^a^	Oral intolerance, dyspnea, and worsening of lesions attributable to mpox (coalescent necrotic lesions)	Hospitalization support; voluntary discharge due to stigma; lost to follow-up after 8 wk of starting ART
Cis man; age, 23 y	HIV diagnosis 1 y ago, stopped ART 16 wk ago. Latest HIV viral load: 12 055 copies/mL. CD4: 255 cells/mm³. Concurrent syphilis and hepatitis C infection	Disseminated pustular lesions with coalescent lesions (120 lesions at its peak), whitish retropharyngeal exudate, odynophagia, fever, headache, myalgia, arthralgia	Day 1: B/F/T^b^	Increase in the number of lesions related to mpox	Support; benzathine penicillin G for syphilis; treatment for hepatitis C deferred until clinical resolution of mpox; outpatient, retention in care

Abbreviations: ART, antiretroviral therapy; IRIS, immune reconstitution inflammatory syndrome.

^a^FTC/TDF (Emtricitabine/Tenofovir Disoproxil Fumarate) + DRV/COBI (Darunavir/Cobicistat).

^b^B/F/T (Bictegravir/Emtricitabine/Tenofovir Alafenamide).

## DISCUSSION

In this small study, no differences were found in the occurrence of mpox-related IRIS between patients with early and late ART initiation with untreated HIV. We identified an IRIS occurrence of 10.5% associated with mpox, which is lower than that in the study by Mitjà et al, the largest published series of mpox in people with advanced HIV infection, where 24.7% of people starting or restarting ART experienced IRIS, among whom 12 of 21 died [2]. Additionally, our sample showed a more favorable outcome. Key differences include the early initiation of antiretroviral treatment by 52.6% in our case, in contrast to 33.3% in the study by Mitjà et al, with a median ART initiation of 21 days. Furthermore, the study revealed a mortality rate of 57.1% attributable to IRIS related to mpox, while we did not report any deaths; however, 1 participant was lost to follow-up.

In a report based on data from the Centers for Disease Control and Prevention, 27 people died, of which 24 were people with HIV and all had CD4+ cell counts <200 [9]. ART was initiated for 19 of the 20 decedents who were not receiving ART, and more than half (54.2%) received steroids for mpox complications or concerns about IRIS. The interval from diagnosis of mpox to initiation of ART was 15 days. In this case series, it is difficult to know if the complications and death were due to the advanced stage of the HIV infection or to IRIS.

We documented 2 cases of mpox exacerbation in individuals who initiated ART; it remains debatable whether this could be considered IRIS or progression of the natural history of mpox. There are several reports of worsening of mpox infection after starting ART, as a regular IRIS presentation [2, 5, 10]. Yet, some studies have indicated that people with a CD4+ cell count <100, a high HIV-1 viral load, >50 skin lesions, and no ART have a more severe and prolonged course of the disease and a higher risk of death [1, 3, 10]. In addition, the impact has been described on the immune response to mpox infection in people with uncontrolled HIV viremia, involving the reduction of not only T lymphocytes but also CD8+ T cells, B cells, and natural killer cells [10, 11]. Clinical worsening after initiation of ART, with a paucity of T lymphocytes, has been observed in biopsies of the lesions [12]. This further explains that the worsening of mpox infection may be due to host conditions and not necessarily to IRIS, where an increase in CD4+ T lymphocytes is expected.

Some limitations of our study need to be highlighted. This retrospective observational study lacks standardized clinical or laboratory follow-up, and the information is limited. The small sample size also limits the findings. The definition for mpox-related IRIS is not standardized or validated in other studies, which represents a major challenge. We advocate for precise differentiation in identifying genuine cases of mpox-related IRIS. We consider that IRIS-related mpox may occur in people with advanced HIV disease who start ART; however, the risk of deterioration and fatal outcomes may be high in patients with very low CD4+ cell counts who initiate ART late [1, 3, 10]. Given the results of initiating ART in other OIs [13–15], such as tuberculosis or pneumocystis, and considering the risk of worse outcomes from not initiating ART, we propose, as with any other OI, early ART initiation in people with mpox infection, especially in the context of advanced HIV disease. This recommendation aligns with utilizing available resources, including approved antivirals, or accessing them through clinical trials, while maintaining close monitoring due to the risk of potentially fatal complications.

## CONCLUSIONS

In this study, we did not find worse outcomes associated with IRIS related to mpox among individuals who initiated antiretroviral treatment early. This suggests that, in the absence of contraindications for starting ART, it should be initiated as soon as possible.

## Notes

***Acknowledgments.*** We thank Dr Andrea González Rodríguez (Clínicas Especializadas Condesa, Mexico City) for assistance in data-sharing agreement administration. We acknowledge the civil society of Mexico for its tireless advocacy for people with HIV during the last multinational outbreak of mpox. We express our gratitude to individuals with mpox and their families who kindly consented to contribute data to enhance understanding of this disease.

***Authors contributions*.** Conceptualization: J. C. R.A. Design of the study: J. C. R.-A. and E. P.-B. Data acquisition: J. I. L.M., G. H.S., A. K. O.L., R. A. C.F., and B. G.F. Preparation of the article: J. C. R.A., E. P.B., and B. C.R. All authors have read and agreed to the published version of the manuscript.

***Financial Support.*** There was no funding source for this study.

***Supplement sponsorship***. This article appears as part of the supplement “Mpox Outbreak 2022 and Beyond: Understanding Transmission to Inform Public Health Responses,” sponsored by the authors.
